# Supplementary material for: A computational analysis of mouse behavior in the sucrose preference test
Source: Nat Commun. 2023 Apr 27;14:2419. doi: 10.1038/s41467-023-38028-0 (PMC10140068; doi:10.1038/s41467-023-38028-0)
Supplement: Supplementary file 1 — Supplementary Information [file 41467_2023_38028_MOESM1_ESM.pdf]

SUPPLEMENTARY FIGURES

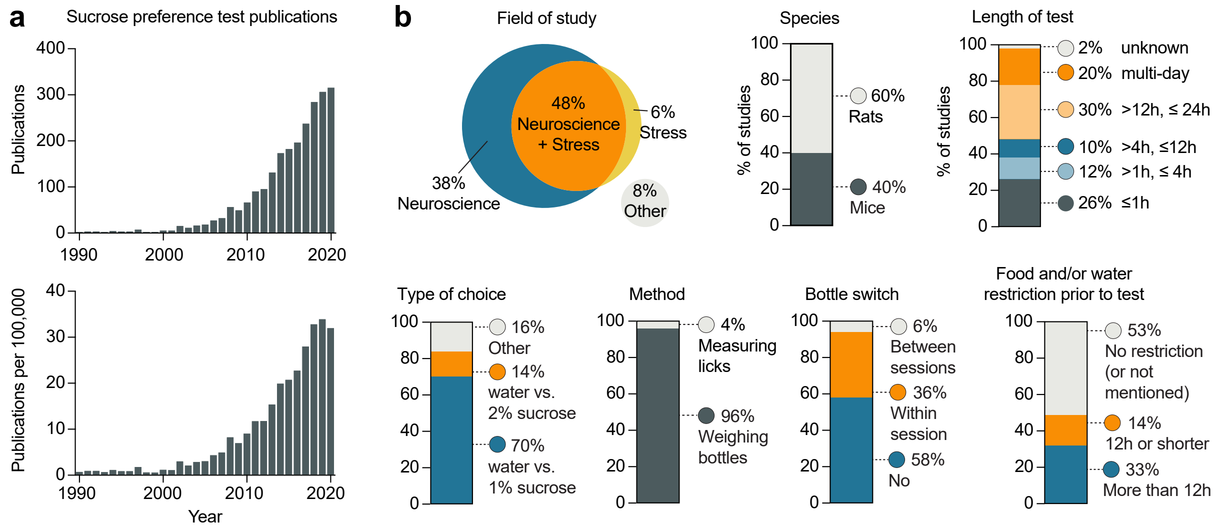

Supplementary Figure 1. Meta-analysis shows high variability in how the SPT is conducted across labs.

(**a**) Number of publications using the sucrose preference test since 1990. Shown is the absolute number of publications (left) and relative to the total number of papers in the Medline database in that year (right). Analysis performed on February 10, 2023 using Pubmed search terms *"sucrose preference" OR ("sucrose" AND "anhedonia").*

(**b**) We performed a systematic meta-analysis on the 50 most recent papers from the Pubmed database that mentioned the SPT in title or abstract, performed on July 13, 2020 using search terms *"sucrose preference" OR ("sucrose" AND "anhedonia")*. Of these papers, 50 (100%) were primary research articles that used some form of the SPT in rodents — 60% of those were performed in rats; 40% in mice. The majority of papers (86%) were in the neuroscience field, directly manipulating neural activity in freely behaving animals or performing post-mortem research on brain tissue. In 54% of all papers, animals were subjected to some sort of stress paradigm; 48% of all papers spanned both neuroscience and stress research. Additional methodological aspects were assessed, including the duration, liquid type, method used to measure sucrose preference, whether sucrose and water bottles were switched within or between sessions, and whether animals were food and/or water restricted prior to the test. Together, this meta-analysis indicates substantial variability in the behavioral protocols used for the SPT.

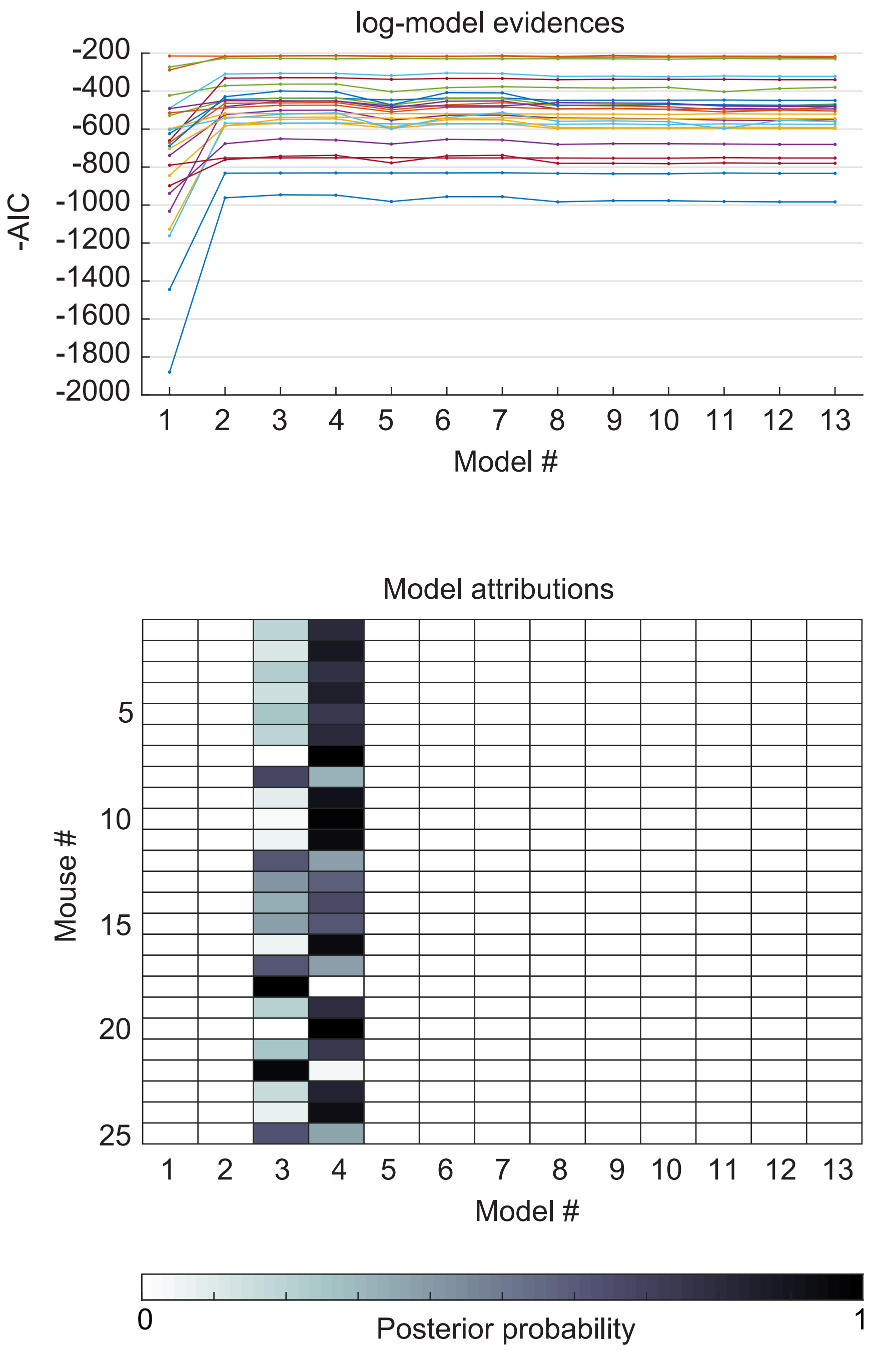

Supplementary Figure 2. Model comparisons.

Additional data on the Bayesian model selection procedure, showing additional measures for each of the 13 models shown in Figure 2a. Top panel shows the Akaike Information Criterion (AIC) for each mouse (indicated by different colors). Bottom panel shows the posterior probability of each model (columns) for each mouse/session (rows).

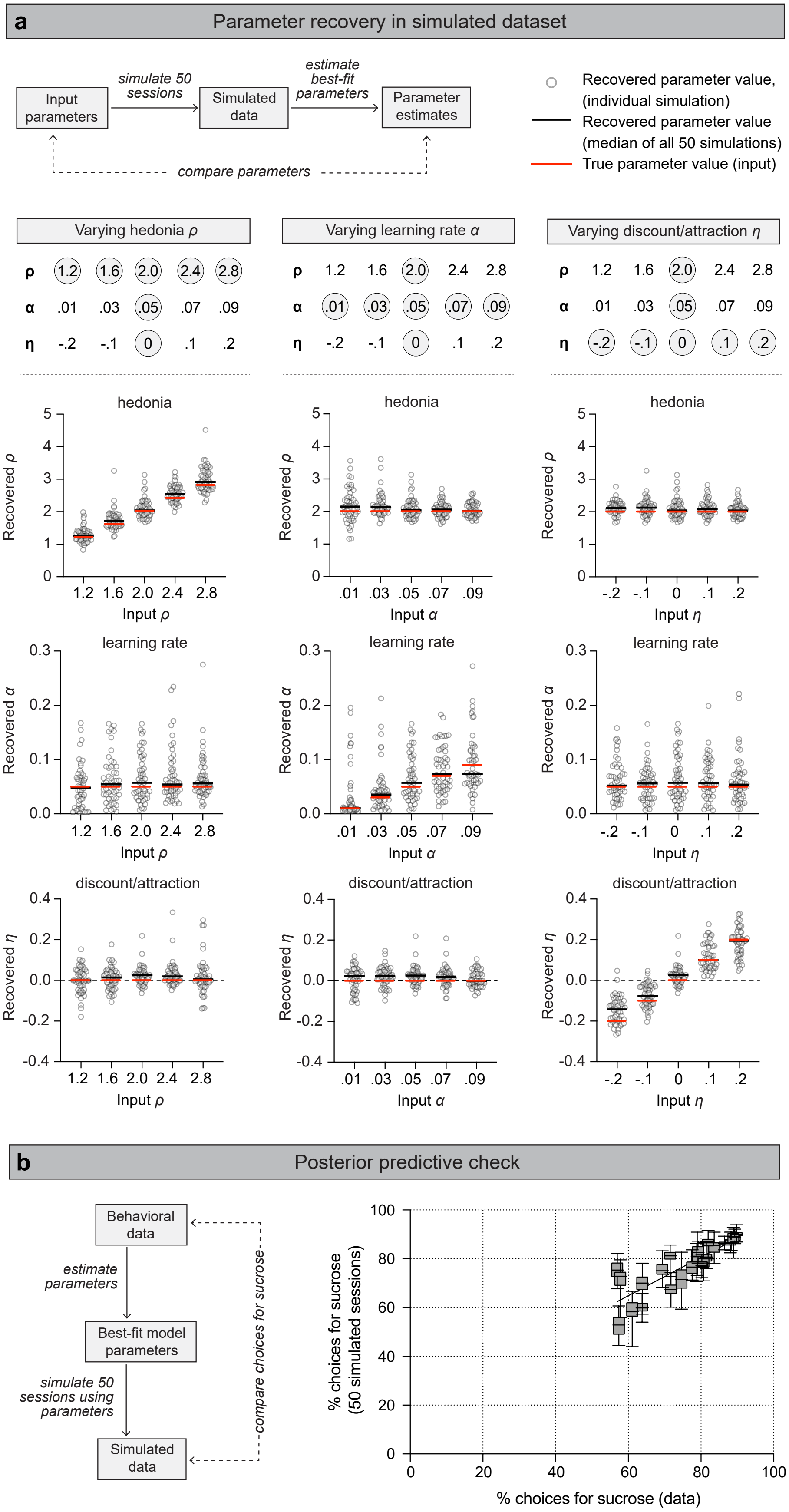
a
Parameter recovery in simulated dataset
Input parameters
simulate 50 sessions
Simulated data
estimate best-fit parameters
Parameter estimates
compare parameters
Recovered parameter value, (individual simulation)
Recovered parameter value (median of all 50 simulations)
True parameter value (input)
Varying hedonia ρ
Varying learning rate α
Varying discount/attraction η
hedonia
learning rate
discount/attraction
Recovered ρ
Recovered α
Recovered η
Input ρ
Input α
Input η
b
Posterior predictive check
Behavioral data
estimate parameters
Best-fit model parameters
simulate 50 sessions using parameters
Simulated data
compare choices for sucrose
% choices for sucrose (50 simulated sessions)
% choices for sucrose (data)

Supplementary Figure 3. Model validations.

(**a**) We validated the computational model in two additional ways. First, we performed a parameter recovery procedure in a simulated dataset. To do this, we simulated SPT sessions in which an agent makes 250 choices for a sucrose or water bottle for a given set of (arbitrary) parameters $\{\rho, \alpha, \eta\}$. Next, we used our maximum likelihood procedure to estimate the best-fit parameters based on the (simulated) raw choice behavior of the agent. We then varied the value of hedonia factor ρ, while keeping learning rate α and discount/attraction parameter η the same. As a result, we were able to retrieve the value of the parameters with high accuracy (first column). Parameter recovery was also successful when varying parameters α or η, while keeping the other two parameters the same (second and third column). This indicates that (i) parameters can be accurately estimated from the raw data, (ii) parameters are independent, and that (iii) different parameters have qualitatively distinct effects on choice behavior in the SPT. For each condition, the parameter recovery procedure was performed 50 times; each simulation is shown as an individual circle. Black horizontal lines indicate the median of those 50 simulations; red horizontal lines indicate the true parameter value (i.e., input values).

(**b**) Second, we performed a posterior predictive check of the model. We used the 25 animals shown in Figures 1 and 2, simulated 50 SPT sessions based on each mouse's $\{\rho, \alpha, \eta\}$ parameter estimates, and compared the number of choices for sucrose in the simulated data with the experimental data. This revealed a high correlation between simulated and experimental data (linear regression, $r = 0.85$, $p < 0.0001$), which also suggests that the three parameters are a good estimator of behavior in the SPT, even in noisy experimental data. Line indicates best-fit linear regression. Boxplots represent median and interquartile range; whiskers indicate the range of 50 simulated sessions per mouse.

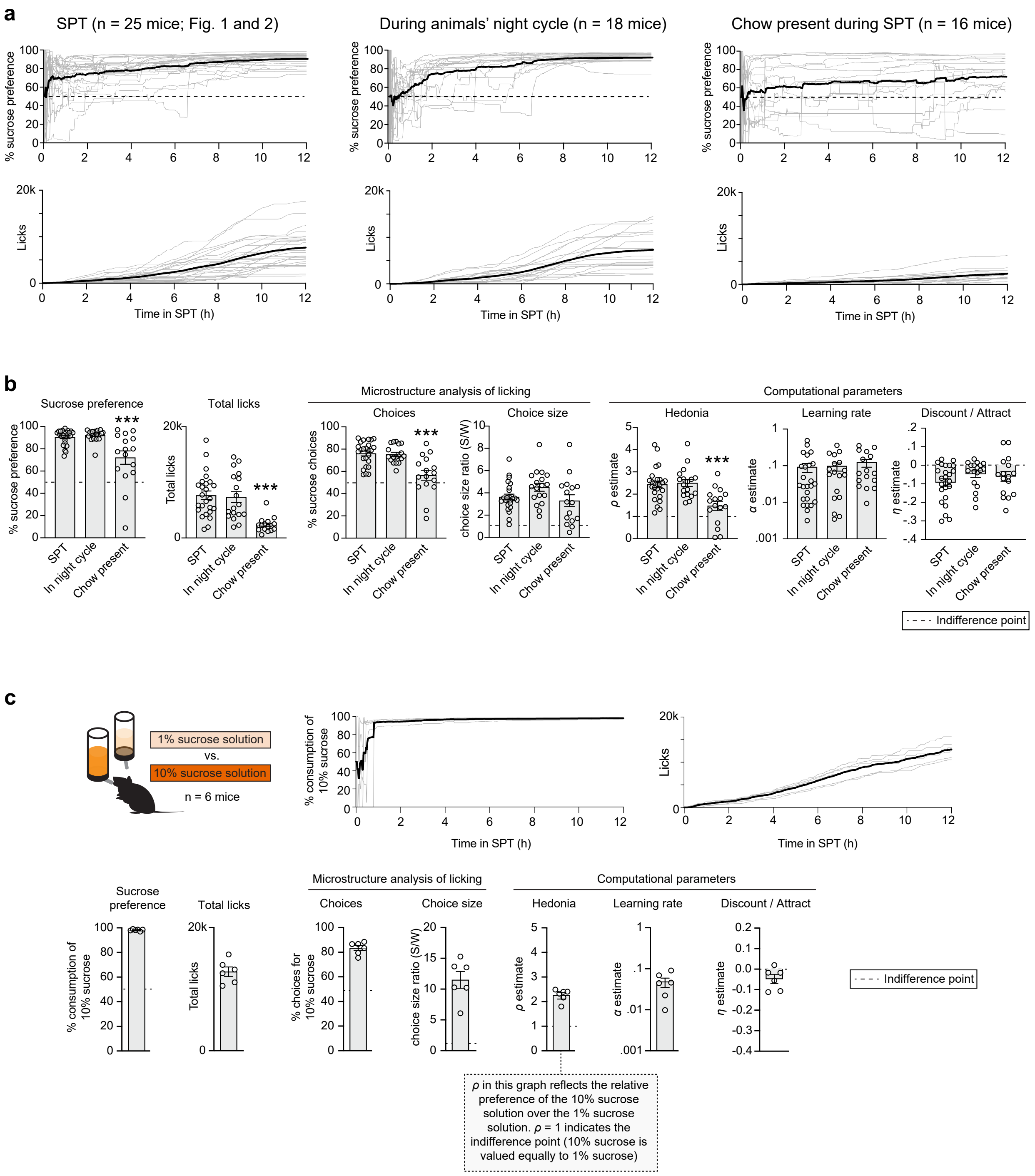

Supplementary Figure 4. Comparison of different experimental conditions for the SPT. (**a**) Development of % sucrose preference (top panels) and total number of licks (bottom panels) over 12 hours for three different experimental conditions. Left: SPT as shown in Figures 1 and 2 (conducted 3 hours during the animals' light cycle and 9 hours during the animals' dark cycle, n

= 25 mice). Middle: SPT that was conducted entirely in the animals' dark cycle (n = 18 mice). Right: SPT that was conduct while animals had *ad libitum* access to regular chow (n = 16 mice). The grey lines indicate individual animals; the black lines indicate the mean. Dashed lines indicate the indifference point (sucrose and water are valued equally).

(**b**) Quantification of behavior for the three different experimental conditions shown in (a). Note that the presence of food reduced sucrose preference and total liquid consumption in the task, as well as the % choices for sucrose through an isolated reduction in hedonia parameter ρ. Circles indicate individual animals. Error bars indicate mean ± SEM. Asterisks indicate statistical significance in one-way ANOVA followed by post-hoc t-tests; * $p < 0.05$, ** $p < 0.01$, *** $p < 0.001$; see Supplementary Table 1 for details on the statistical analysis. Dashed lines indicate the indifference point (sucrose and water are valued equally).

(**c**) Behavior in a two-bottle choice test with 1% and 10% sucrose solutions (n = 6 mice). Top panels show the development of preference for the 10% sucrose solution and total consumption over time (grey lines indicate individual animals; black lines indicate the mean). Bottom panels show quantification of behavior; $\rho > 1$ indicates that the model fitting procedure can accurately assess a preference for the 10% sucrose solution over the 1% sucrose solution. Error bars indicate mean ± SEM. Dashed lines indicate the indifference point (10% and 1% sucrose are valued equally).

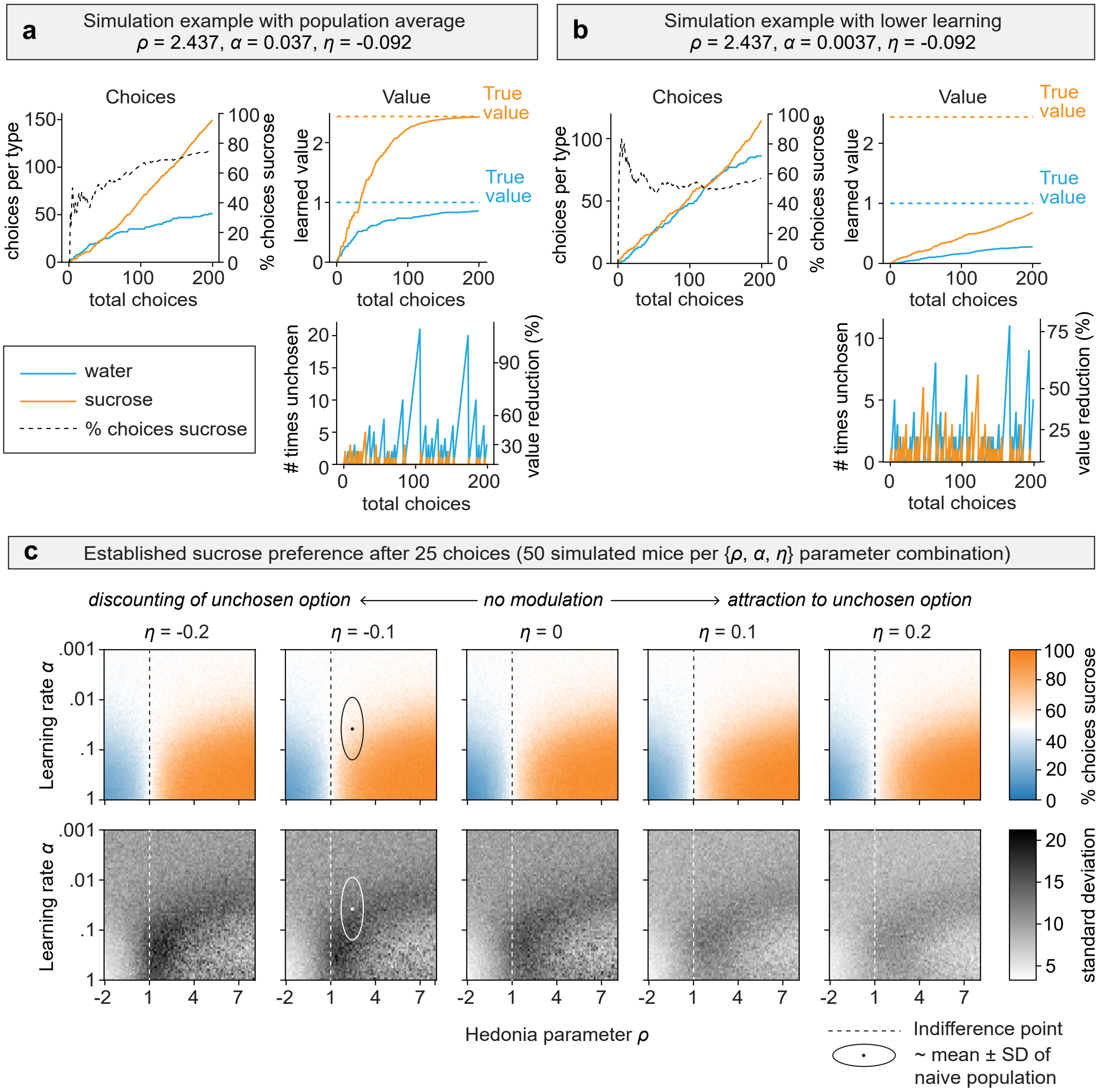

Supplementary Figure 5. Model simulations.

(**a**) Sample simulation of an agent making 200 choices in the SPT. Choices were simulated using the average parameter values of the group of mice shown in Figure 2d.

(**b**) Same simulation, but with a learning rate *a* that is a factor 10 lower. With this lower learning rate, it takes longer before the agent starts developing a preference for the sucrose bottle.

(**c**) Simulated choices for sucrose for a larger combination of parameter values {*ρ*, *α*, *η*}. Shown are heatmaps with the average % choices for sucrose (top) and standard deviation (bottom) after 25 choices for a colony of 50 mice per *{ρ, α, η}* parameter combination. Each heatmap shows the

change in % choices for sucrose per $\{\rho, \alpha\}$ combination; different columns represent different values of η. Hedonia parameter ρ and learning rate α together mainly establish the % choices for sucrose in the SPT. The dynamic range for the % choices for sucrose is approximately between $0.01 < \alpha < 0.1$ and $1 < \rho < 4$, with the % choices plateauing beneath and above those values. This means that any changes to learning rate α and hedonia parameter ρ outside of this range will not be reflected in the % choices for sucrose, possibly masking some cases of anhedonia or learning deficits when studying conventional outcome measures of the SPT. Thus, parameter estimation may reveal behavioral changes in experimental data that may not be detected otherwise. Interestingly, although discounting/attraction parameter η does not directly influence the average % choices for sucrose (i.e., compare 5 different columns), it does provide an additional source of inter-animal variation, with more negative values of η being associated with more variation (i.e., compare 5 different columns of the black-and-white heat maps). Dashed line indicates indifference point ($\rho = 1$, i.e., sucrose has same value as water). Circle depicts the approximate mean ± 1 S.D. from the population distribution shown in Figure 2d.

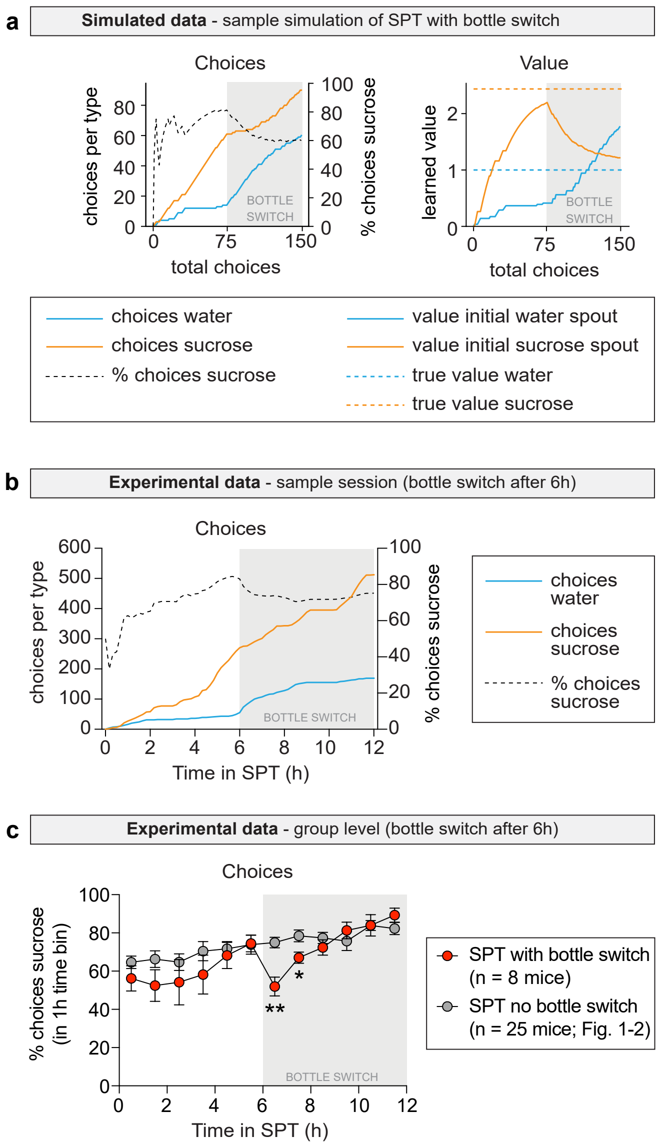

Supplementary Figure 6. SPT with switch of sucrose and water bottles.

(**a**) Sample simulation (as shown in Supplementary Fig. 5a), but for an SPT that involved switching the sucrose and water bottles after 75 choices.

(**b**) Example of an experimental SPT session in which the sucrose and water bottles were switched after 6 hours.

(**c**) Group average of the % choices for sucrose for an experiment in which the water and sucrose bottles were switched after 6 hours (n = 8 mice). Switching these bottles induced a significant reduction in % choices for sucrose for up to 2 hours. Error bars indicate mean ± SEM. Asterisks indicate statistical significance in unpaired t-tests after a 2-way repeated measures ANOVA; * $p < 0.05$, ** $p < 0.01$; see Supplementary Table 1 for additional details on the statistical analysis.

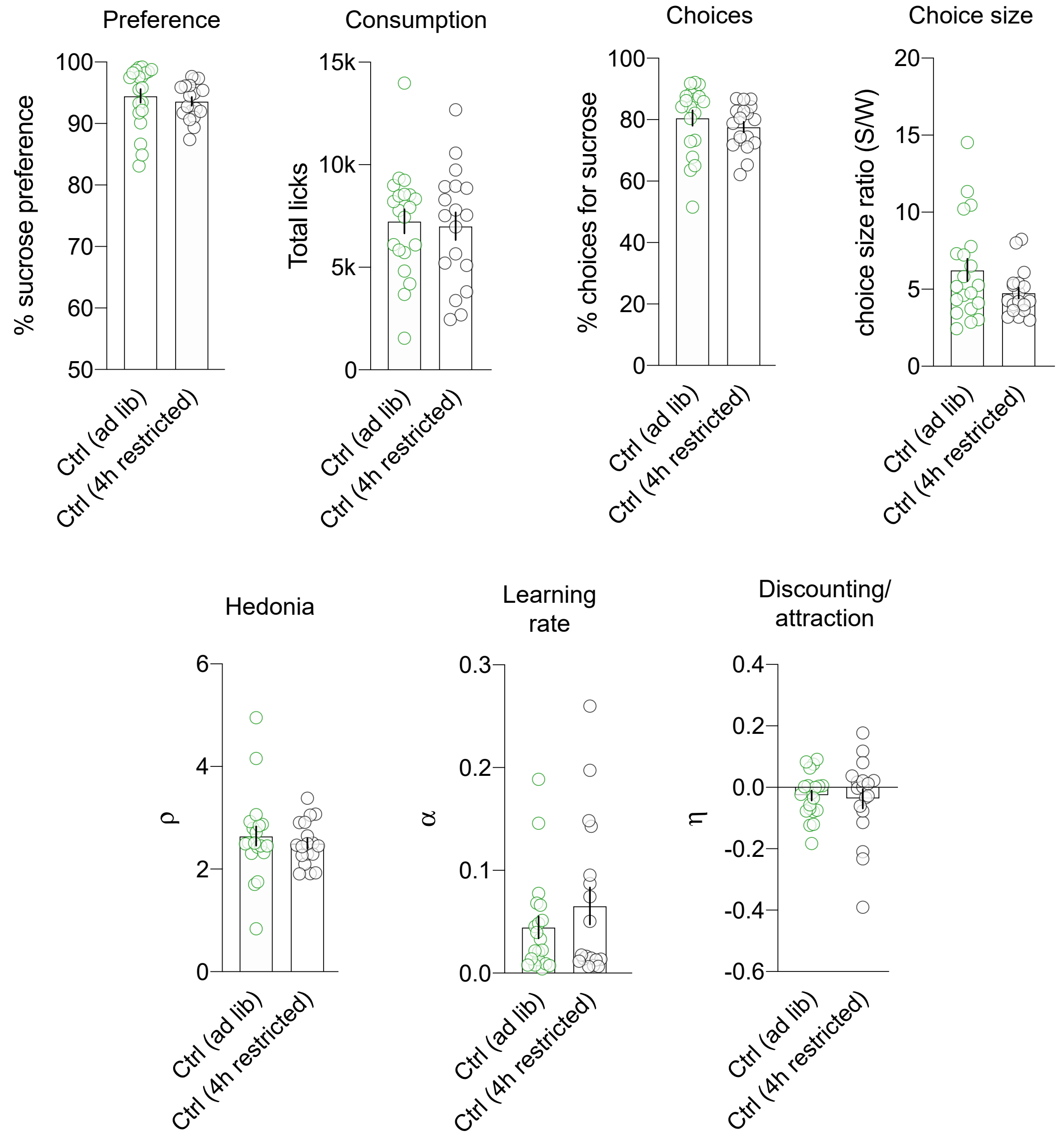

Supplementary Figure 7. No significant differences between the two control groups in the acute stress experiment.

Two different control groups were used for the acute stress experiment (Fig. 3b) to control for the fact that animals had no access to food and water in the restrainer. However, for none of the outcome measures there was a significant difference between the *ad libitum*-fed and food-restricted control groups. As such, these control groups were merged in Figure 3b. Groups were compared using unpaired t-tests; for additional details on the statistical analysis, see Supplementary Table 1.

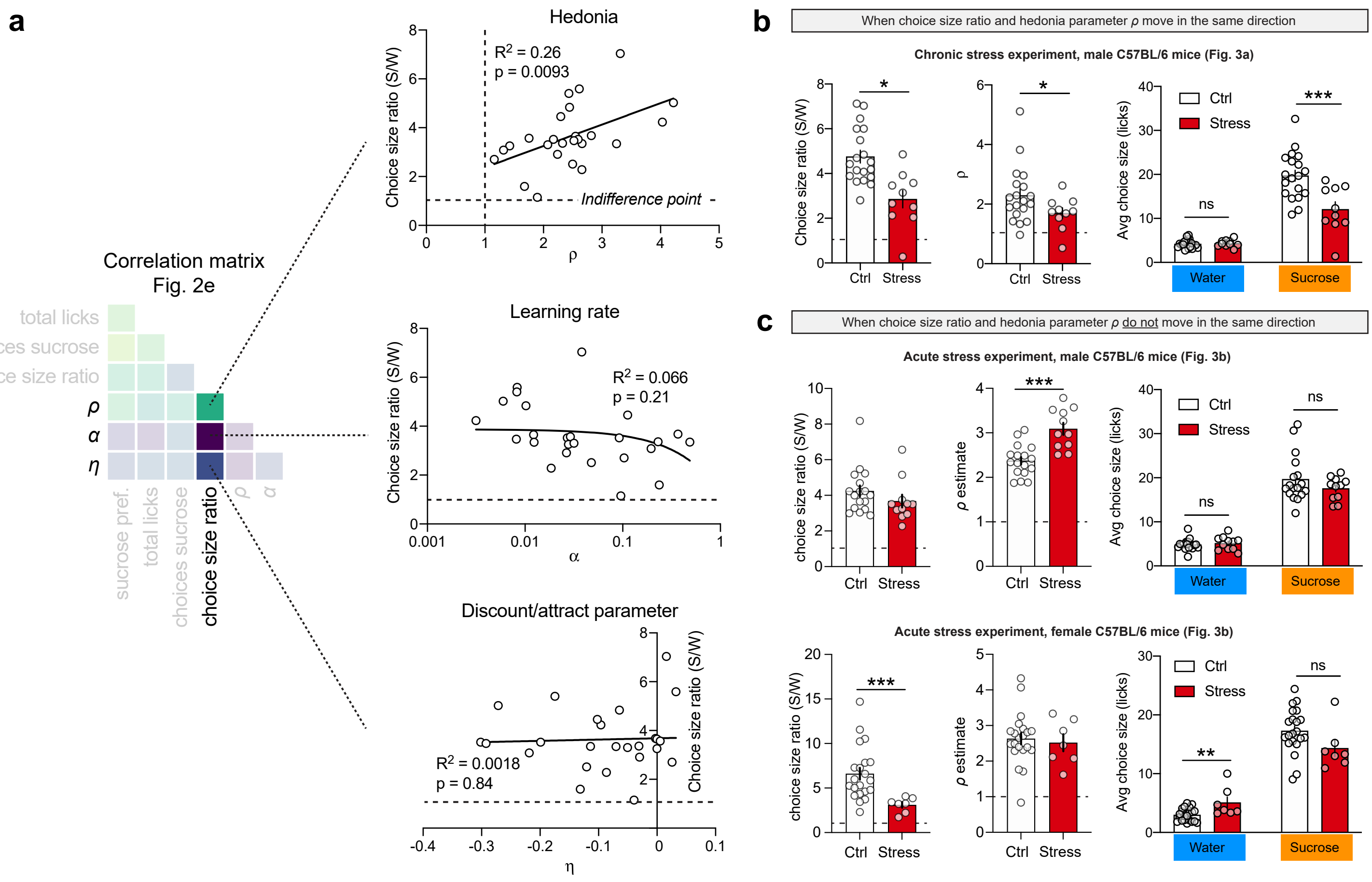

Supplementary Figure 8. Choice size ratio as a proxy for hedonia.

(**a**) Schematic showing the underlying regression plots for choice size ratio of the correlation matrix of Figure 2e. Choice size ratio positively correlates with hedonia parameter ρ, albeit with a low R^2 of 26% (linear regression).

(**b**) We also analyzed three experiments in which we observed a reduction in choice size ratio in the experimental versus control condition. In only 1 out of those 3 experiments, a reduction in choice size ratio coincided with a reduction in hedonia parameter ρ. In this case, the reduction in choice size ratio was driven by a reduction in the average number of licks per choice for sucrose, and not for water. Error bars indicate mean ± SEM. Asterisks indicate statistical significance in unpaired t-tests (in the right panel an unpaired t-test after a 2-way repeated measures ANOVA); * $p < 0.05$, *** $p < 0.001$; see Supplementary Table 1 for additional details on the statistical analysis.

(**c**) In the other case, in which a reduction in choice size ratio did not coincide with a reduction in hedonia parameter ρ, the reduction in choice size ratio was driven by an increased number of

licks per choice for water. Together, these data suggest that choice size ratio can be used as a proxy for hedonia parameter ρ only if the change in choice size ratio is driven by an isolated change in the average sucrose choice size (i.e., without a change in water choice size). Asterisks indicate statistical significance in unpaired t-tests (in the right panels an unpaired t-test after a 2-way repeated measures ANOVA); * $p < 0.05$, ** $p < 0.01$, *** $p < 0.001$; see Supplementary Table 1 for details on the statistical analysis.

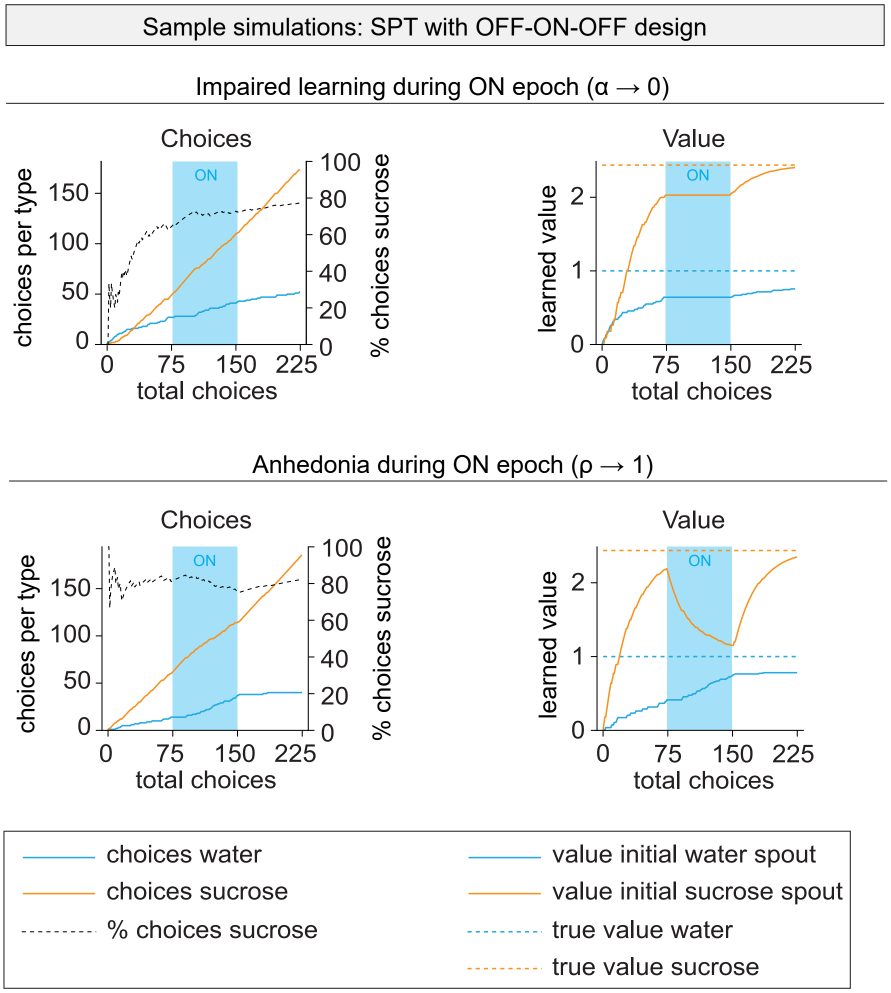

Supplementary Figure 9. Sample simulations for optogenetic experiments.

Sample simulations for the optogenetic experiments (Fig. 4d). Simulations were performed with the average of the best-fit model parameters (Fig. 2d); during the ON epoch (between choices 75-150), a learning impairment ($a \longrightarrow 0$, top panels) or anhedonia ($\rho \longrightarrow 1$, bottom panels) was induced. This shows that a learning impairment temporarily stalls increments in bottle value, while anhedonia actively reduces the value of the sucrose bottle towards that of the water bottle. This explains why, after learning has been established, acute anhedonia reduces the % choices for sucrose, while a learning deficit does not.

SUPPLEMENTARY TABLE 1

Figure	n	Sex	Test used	Test statistics	P value
1c, number of choices	25 mice	Mixed	Paired t-test, two tailed	t(24) = 6.227	**P < 0.0001**
1c, licks per choice	25 mice	Mixed	Paired t-test, two tailed	t(24) = 11.81	**P < 0.0001**
2a	25 mice	Mixed	Bayesian model selection	Model / Aggregate AIC<br>1 / 18629,02<br>2 / 12719,06<br>3 / 12462,01<br>**4 / 12451,05**<br>5 / 13076,99<br>6 / 12615,61<br>7 / 12563,59<br>8 / 12994,69<br>9 / 12975,95<br>10 / 13006,12<br>11 / 13086,48<br>12 / 13057,85<br>13 / 13033,71	
2f, preference	Repeated measures in 16 mice	Mixed	Paired t-test, two tailed	**t(15) = 5.082**	**P = 0.0001**
2f, choices	Repeated measures in 16 mice	Mixed	Paired t-test, two tailed	**t(15) = 4.358**	**P = 0.0006**
2f, choice size	Repeated measures in 16 mice	Mixed	Paired t-test, two tailed	**t(15) = 2.503**	**P = 0.0244**
2f, hedonia	Repeated measures in 16 mice	Mixed	Paired t-test, two tailed	**t(15) = 4.352**	**P = 0.0006**
2f, learning rate	Repeated measures in 16 mice	Mixed	Paired t-test, two tailed	t(15) = 1.845	P = 0.0849
2f, discount/attract	Repeated measures in 16 mice	Mixed	Paired t-test, two tailed	t(15) = 1.444	P = 0.1694
3a (top), preference	19 vs. 10 mice	Male	Mann-Whitney test, one tailed	U = 35	**P = 0.0024**
3a (top), licks	19 vs. 10 mice	Male	Unpaired t-test with Welch correction (for unequal sd), two tailed	t(25.33) = 5.581	**P < 0.0001**
3a (top), choices	19 vs. 10 mice	Male	Unpaired t-test, two tailed	t(27) = 1.750	**P = 0.0458**
3a (top), choice size	19 vs. 10 mice	Male	Unpaired t-test, two tailed	t(27) = 3.894	**P = 0.0006**
3a (top), hedonia	19 vs. 10 mice	Male	Unpaired t-test with Welch correction (for unequal sd), two tailed	t(26.64) = 2.103	**P = 0.0450**

Figure	n	Sex	Test used	Test statistics	P value
3a (top), learning rate	19 vs. 10 mice	Male	Mann-Whitney test, two tailed	U = 92	P = 0.9103
3a (top), discount/attract	19 vs. 10 mice	Male	Unpaired t-test, two tailed	t(27) = 0.007	P = 0.9948
3a (bottom), preference	15 vs. 15 mice	Female	Mann-Whitney test, one-tailed	U = 53	**P = 0.0064**
3a (bottom), licks	15 vs. 15 mice	Female	Unpaired t-test, two tailed	t(28) = 3.974	**P = 0.0005**
3a (bottom), choices	15 vs. 15 mice	Female	Unpaired t-test, two tailed	t(28) = 2.803	**P = 0.0045**
3a (bottom), choice size	15 vs. 15 mice	Female	Unpaired t-test, two tailed	t(28) = 0.2859	P = 0.7771
3a (bottom), hedonia	15 vs. 15 mice	Female	Unpaired t-test with Welch correction (for unequal sd), two tailed	t(17.72) = 0.3470	P = 0.7326
3a (bottom), learning rate	15 vs. 15 mice	Female	Mann-Whitney test, two-tailed	U = 106	P = 0.8063
3a (bottom), discount/attract	15 vs. 15 mice	Female	Unpaired t-test, two tailed	t(28) = 0.4023	P = 0.6905
3b (top), preference	17 vs. 11 mice	Male	Unpaired t-test, one tailed	t(26) = 0.5764	P = 0.2846
3b (top), licks	17 vs. 11 mice	Male	Unpaired t-test, two tailed	t(26) = 0.5579	P = 0.5817
3b (top), choices	17 vs. 11 mice	Male	Unpaired t-test, two tailed	t(26) = 1.544	P = 0.1346
3b (top), choice size	17 vs. 11 mice	Male	Unpaired t-test with Welch correction (for unequal sd), two tailed	t(1.182) = 22.71	P = 0.2495
3b (top), hedonia	17 vs. 11 mice	Male	Unpaired t-test, two tailed	t(26) = 4.731	**P < 0.0001**
3b (top), learning rate	17 vs. 11 mice	Male	Unpaired t-test, two tailed	t(26) = 2.286	**P = 0.0307**
3b (top), discount/attract	17 vs. 11 mice	Male	Mann-Whitney test, two tailed	U = 89	P = 0.8533
3b (bottom), preference	21 vs. 7 mice	Female	Mann-Whitney test, one tailed	U = 28	**P = 0.0072**
3b (bottom), licks	21 vs. 7 mice	Female	Unpaired t-test, two tailed	t(26) = 0.5440	P = 0.5911
3b (bottom), choices	21 vs. 7 mice	Female	Mann-Whitney test, two tailed	U = 44	P = 0.1259

Figure	n	Sex	Test used	Test statistics	P value
3b (bottom), choice size	21 vs. 7 mice	Female	Unpaired t-test with Welch correction (for unequal sd), two tailed	t(25.76) = 4744	**P < 0.0001**
3b (bottom), hedonia	21 vs. 7 mice	Female	Unpaired t-test, two tailed	t(26) = 0.3622	P = 0.7201
3b (bottom), learning rate	21 vs. 7 mice	Female	Unpaired t-test on log-transformed data, two tailed	t(26) = 0.2868	P = 0.7765
3b (bottom), discount/attract	21 vs. 7 mice	Female	Unpaired t-test with Welch correction (for unequal sd), two tailed	t(8.063) = 0.5428	P = 0.6019
4c, sucrose preference (eNPHR3-eYFP)	Repeated measures in 5 mice	Mixed	Two-way RM ANOVA	Epoch: F(2,8) = 2.379<br>Stim: F(2,8) = 8.587<br>Interaction: F(4,16) = 0.3317	P = 0.1546<br>**P = 0.0102**<br>P = 0.8526
			Post-hoc test, stimulation session vs OFF-OFF-OFF session	Epoch 1<br>OFF-ON-OFF: t(16) = 0.68<br>ON-OFF-OFF: t(16) = 2.8<br><br>Epoch 2<br>OFF-ON-OFF: t(16) = 0.018<br>ON-OFF-OFF: t(16) = 2.2<br><br>Epoch 3<br>OFF-ON-OFF: t(16) = 0.092<br>ON-OFF-OFF: t(16) = 2.0	 P = 0.5026<br>**P = 0.0140**<br><br><br>P = 0.9862<br>**P = 0.0436**<br><br><br>P = 0.9280<br>P = 0.0627
4c, choices for sucrose (eNPHR3-eYFP)	Repeated measures in 5 mice	Mixed	Two-way RM ANOVA	Epoch: F(2,8) = 1.367<br>Stim: F(2,8) = 7.637<br>Interaction: F(4,16) = 0.1324	P = 0.3086<br>**P = 0.0140**<br>P = 0.9682
			Post-hoc test, stimulation session vs OFF-OFF-OFF session	Epoch 1<br>OFF-ON-OFF: t(16) = 0.10<br>ON-OFF-OFF: t(16) = 3.1<br><br>Epoch 2<br>OFF-ON-OFF: t(16) = 0.53<br>ON-OFF-OFF: t(16) = 3.4<br><br>Epoch 3<br>OFF-ON-OFF: t(16) = 0.11<br>ON-OFF-OFF: t(16) = 2.5	 P = 0.9204<br>**P = 0.0071**<br><br><br>P = 0.6007<br>**P = 0.0034**<br><br><br>P = 0.9125<br>**P = 0.0244**
4c, total licks (eNPHR3-eYFP)	Repeated measures in 5 mice	Mixed	Two-way RM ANOVA	Epoch: F(2,8) = 57.14<br>Stim: F(2,8) = 0.3659<br>Interaction: F(4,16) = 0.4840	P = 0.0015<br>P = 0.6690<br>P = 0.6286
4c, sucrose preference (eYFP)	Repeated measures in 6 mice	Mixed	Two-way RM ANOVA	Epoch: F(2,10) = 0.2264<br>Stim: F(2,10) = 0.04522<br>Interaction: F(4,20) = 0.7070	P = 0.8014<br>P = 0.9560<br>P = 0.5965

Figure	n	Sex	Test used	Test statistics	P value
4c, choices for sucrose (eYFP)	Repeated measures in 6 mice	Mixed	Two-way RM ANOVA	Epoch: $F(2,10) = 2.173$<br>Stim: $F(2,10) = 0.1224$<br>Interaction: $F(4,20) = 1.250$	$P = 0.1646$<br>$P = 0.8861$<br>$P = 0.3221$
4c, total licks (eYFP)	Repeated measures in 6 mice	Mixed	Two-way RM ANOVA	Epoch: $F(2,10) = 96.54$<br>Stim: $F(2,10) = 1.897$<br>Interaction: $F(4,20) = 0.8267$	$P < 0.0001$<br>$P = 0.2129$<br>$P = 0.4718$
Suppl. Fig. 3b	25 mice (50 simulations each)	Mixed	Linear regression	$r = 0.853$<br>$R^2 = 0.728$	**$P < 0.0001$**
Suppl. Fig. 4b, sucrose preference	n = 25 mice (normal)<br>n = 18 (night cycle)<br>n = 16 (chow present)	Mixed	One-way ANOVA	$F(2,56) = 11.72$	**$P < 0.0001$**
			Post-hoc tests (special test vs. normal SPT)	Night: $t(56) = 0.3935$<br>Chow: $t(56) = 4.254$	$P = 0.6954$<br>**$P < 0.0001$**
Suppl. Fig. 4b, total licks	n = 25 mice (normal)<br>n = 18 (night cycle)<br>n = 16 (chow present)	Mixed	One-way ANOVA	$F(2,56) = 12.93$	**$P < 0.0001$**
			Post-hoc tests (special test vs. normal SPT)	Night: $t(56) = 0.2979$<br>Chow: $t(56) = 4.763$	$P = 0.7669$<br>**$P < 0.0001$**
Suppl. Fig. 4b, choices	n = 25 mice (normal)<br>n = 18 (night cycle)<br>n = 16 (chow present)	Mixed	One-way ANOVA	$F(2,56) = 14.08$	**$P < 0.0001$**
			Post-hoc tests (special test vs. normal SPT)	Night: $t(56) = 0.1893$<br>Chow: $t(56) = 4.928$	$P = 0.8505$<br>**$P < 0.0001$**
Suppl. Fig. 4b, choice size	n = 25 mice (normal)<br>n = 18 (night cycle)<br>n = 16 (chow present)	Mixed	One-way ANOVA	$F(2,56) = 2.639$	$P = 0.0803$
Suppl. Fig. 4b, hedonia	n = 25 mice (normal)<br>n = 18 (night cycle)<br>n = 16 (chow present)	Mixed	One-way ANOVA	$F(2,56) = 10.48$	**$P = 0.0001$**
			Post-hoc tests (special test vs. normal SPT)	Night: $t(56) = 0.2781$<br>Chow: $t(56) = 4.068$	$P = 0.7820$<br>P = **0.0001**
Suppl. Fig. 4b, learning rate	n = 25 mice (normal)<br>n = 18 (night cycle)<br>n = 16 (chow present)	Mixed	Kruskal-Wallis test	K-W statistic: 2.361	$P = 0.3072$
Suppl. Fig. 4b, discount/attract	n = 25 mice (normal)<br>n = 18 (night cycle)<br>n = 16 (chow present)	Mixed	One-way ANOVA	$F(2,56) = 1.290$	$P = 0.2834$
Suppl. Fig. 6c	25 vs 8 mice	Mixed	Two-way RM ANOVA on second half of test	Epoch: $F(3.5, 98.4) = 96.54$<br>SPT type: $F(1,31) = 0.9353$<br>Interaction: $F(5,139) = 5.031$	**$P < 0.0001$**<br>$P = 0.3410$<br>**$P = 0.0003$**

Figure	n	Sex	Test used	Test statistics	P value
			Post-hoc tests	7th h: t(11.98) = 4.063<br>8th h: t(23.67) = 2.674<br>9th h: t(15.33) = 1.001<br>10th h: t(24.17) = 0.8585<br>11th h: t(8.730) = 0.01119<br>12th h: t(10.89) = 1.431	**P = 0.0016**<br>**P = 0.0134**<br>P = 0.3324<br>P = 0.3990<br>P = 0.9913<br>P = 0.1805
Suppl. Fig. 7, preference	20 vs 18 mice	Mixed	Unpaired t-test (ad lib ctrl. versus food restricted ctrl), two tailed	t(36) = 0.6673	P = 0.5088
Suppl. Fig. 7, total licks	20 vs 18 mice	Mixed	Unpaired t-test (ad lib ctrl. versus food restricted ctrl), two tailed	t(36) = 0.2626	P = 0.7944
Suppl. Fig. 7, choices	20 vs 18 mice	Mixed	Mann-Whitney test (ad lib ctrl. versus food restricted ctrl), two tailed	U = 124	P = 0.1052
Suppl. Fig. 7, choice size	20 vs 18 mice	Mixed	Unpaired t-test on log transformed data (ad lib ctrl. versus food restricted ctrl), two tailed	t(36) = 1.488	P = 0.1454
Suppl. Fig. 7, hedonia	20 vs 18 mice	Mixed	Mann-Whitney test (ad lib ctrl. versus food restricted ctrl), two tailed	U = 156	P = 0.4965
Suppl. Fig. 7, learning rate	20 vs 18 mice	Mixed	Mann-Whitney test (ad lib ctrl. versus food restricted ctrl), two tailed	U = 169	P = 0.7616
Suppl. Fig. 7, discount/attract	20 vs 18 mice	Mixed	Unpaired t-test (ad lib ctrl. versus food restricted ctrl), two tailed	t(36) = 0.2978	P = 0.7676
Suppl. Fig. 8b, hedonia	Same as Fig. 3a (top row)				
Suppl. Fig. 8b, choice size ratio					
Suppl. Fig. 8b, choice sizes		Male	Two-way RM ANOVA	Liquid: F(1,27) = 138.6<br>Stress: F(1,27) = 10.93<br>Liquid * stress interaction: F(1,27) = 15.35	**P < 0.0001**<br>**P = 0.0027**<br>**P = 0.0005**
			Post-hoc tests Stress vs. control	Water: t(27) = 0.3266<br>Sucrose: t(27) = 3.635	P = 0.7465<br>**P = 0.0012**

Figure	n	Sex	Test used	Test statistics	P value
Suppl. Fig. 8c, male data, hedonia	Same as Fig. 3b (top row)				
Suppl. Fig. 8c, male date, choice size ratio					
Suppl. Fig. 8c, male data, choice sizes	17 vs 11 mice	Male	Two-way RM ANOVA	Liquid: F(1,26) = 310.4<br>Stress: F(1,26) = 0.7365<br>Liquid * stress interaction: F(1,26) = 2.457	**P < 0.0001**<br>P = 0.3986<br>P = 0.1291
Suppl. Fig. 8c, female data, hedonia	Same as Fig. 3b (bottom row)				
Suppl. Fig. 8c, female date, choice size ratio					
Suppl. Fig. 8c, female data, choice sizes	21 vs 7 mice	Female	Two-way RM ANOVA	Liquid: F(1,26) = 218.6<br>Stress: F(1,26) = 0.1753<br>Liquid * stress interaction: F(1,26) = 10.00	**P < 0.0001**<br>P = 0.6789<br>**P = 0.0039**
			Post-hoc tests Stress vs. control	Water: t(26) = 3.132<br>Sucrose: t(26) = 1.766	**P = 0.0043**<br>**P = 0.0892**
