# Supplementary material for: A computational analysis of mouse behavior in the sucrose preference test
Source: Nat Commun. 2023 Apr 27;14:2419. doi: 10.1038/s41467-023-38028-0 (PMC10140068; doi:10.1038/s41467-023-38028-0)
Supplement: Supplementary file 3 — Description of Additional Supplementary Files [file 41467_2023_38028_MOESM3_ESM.pdf]

Description of Additional Supplementary Files

File Name: Supplementary Movie 1
Description: The video shows simultaneous behavioral and computational analysis of a sample mouse in the sucrose preference test.
