# Supplementary material for: A computational analysis of mouse behavior in the sucrose preference test
Source: Nat Commun. 2023 Apr 27;14:2419. doi: 10.1038/s41467-023-38028-0 (PMC10140068; doi:10.1038/s41467-023-38028-0)
Supplement: Supplementary file 5 — Reporting Summary [file 41467_2023_38028_MOESM5_ESM.pdf]

nature portfolio

Corresponding author(s): Stephan Lammel

Last updated by author(s): Apr 4, 2023

Reporting Summary

Nature Portfolio wishes to improve the reproducibility of the work that we publish. This form provides structure for consistency and transparency in reporting. For further information on Nature Portfolio policies, see our Editorial Policies and the Editorial Policy Checklist.

Statistics

For all statistical analyses, confirm that the following items are present in the figure legend, table legend, main text, or Methods section.

n/a	Confirmed	
☐	☒	The exact sample size (*n*) for each experimental group/condition, given as a discrete number and unit of measurement
☐	☒	A statement on whether measurements were taken from distinct samples or whether the same sample was measured repeatedly
☐	☒	The statistical test(s) used AND whether they are one- or two-sided<br>*Only common tests should be described solely by name; describe more complex techniques in the Methods section.*
☐	☒	A description of all covariates tested
☐	☐	A description of any assumptions or corrections, such as tests of normality and adjustment for multiple comparisons
☐	☒	A full description of the statistical parameters including central tendency (e.g. means) or other basic estimates (e.g. regression coefficient) AND variation (e.g. standard deviation) or associated estimates of uncertainty (e.g. confidence intervals)
☐	☒	For null hypothesis testing, the test statistic (e.g. *F*, *t*, *r*) with confidence intervals, effect sizes, degrees of freedom and *P* value noted<br>*Give P values as exact values whenever suitable.*
☐	☒	For Bayesian analysis, information on the choice of priors and Markov chain Monte Carlo settings
☒	☐	For hierarchical and complex designs, identification of the appropriate level for tests and full reporting of outcomes
☐	☒	Estimates of effect sizes (e.g. Cohen's *d*, Pearson's *r*), indicating how they were calculated

Our web collection on statistics for biologists contains articles on many of the points above.

Software and code

Policy information about availability of computer code

Data collection	Zen Software 2.3 (Zeiss) was used for acquiring epifluorescence images.
Data analysis	Comparative statistical tests were performed in GraphPad Prism 8; linear regressions shown in Figure 2 were performed in Python (statsmodels). Python package SweetiePy, including data of a sample session, is available at www.github.com/jeroenphv/SweetiePy

For manuscripts utilizing custom algorithms or software that are central to the research but not yet described in published literature, software must be made available to editors and reviewers. We strongly encourage code deposition in a community repository (e.g. GitHub). See the Nature Portfolio guidelines for submitting code & software for further information.

Data

Policy information about availability of data

All manuscripts must include a data availability statement. This statement should provide the following information, where applicable:

- Accession codes, unique identifiers, or web links for publicly available datasets
- A description of any restrictions on data availability
- For clinical datasets or third party data, please ensure that the statement adheres to our policy

Source data are provided with this paper. Any additional data that support the findings of this study are available from the corresponding author upon reasonable request.

Human research participants

Policy information about studies involving human research participants and Sex and Gender in Research.

Reporting on sex and gender	n/a
Population characteristics	n/a
Recruitment	n/a
Ethics oversight	n/a

Note that full information on the approval of the study protocol must also be provided in the manuscript.

Field-specific reporting

Please select the one below that is the best fit for your research. If you are not sure, read the appropriate sections before making your selection.

☒ Life sciences ☐ Behavioural & social sciences ☐ Ecological, evolutionary & environmental sciences

For a reference copy of the document with all sections, see nature.com/documents/nr-reporting-summary-flat.pdf

Life sciences study design

All studies must disclose on these points even when the disclosure is negative.

Sample size	No statistical methods were used to predetermine sample size, which were based on work in previous publications (e.g., Cerniauskas et al., 2019; Neuron).
Data exclusions	No data exclusions.
Replication	No formal within-study replication was performed, because results were largely consistent with previous observations of chronic and acute stress on sucrose preference behavior as well as optogenetic manipulations of PFC neurons.
Randomization	Animals were randomly assigned stress or control group. For repeated measures in the same animals (Fig. 4) the order of the sessions was randomized between animals.
Blinding	For experiments shown in Figure 4, eNpHR or eYFP expression patterns were verified by an experimenter blinded to behavioral outcome. For all other experiments, analysis was fully automated and no blinding was performed.

Reporting for specific materials, systems and methods

We require information from authors about some types of materials, experimental systems and methods used in many studies. Here, indicate whether each material, system or method listed is relevant to your study. If you are not sure if a list item applies to your research, read the appropriate section before selecting a response.

Materials & experimental systems

n/a	Involved in the study	
☒	☐	Antibodies
☒	☐	Eukaryotic cell lines
☒	☐	Palaeontology and archaeology
☐	☒	Animals and other organisms
☒	☐	Clinical data
☒	☐	Dual use research of concern

Methods

n/a	Involved in the study	
☒	☐	ChIP-seq
☒	☐	Flow cytometry
☒	☐	MRI-based neuroimaging

Animals and other research organisms

Policy information about studies involving animals; ARRIVE guidelines recommended for reporting animal research, and Sex and Gender in Research

Laboratory animals	C57Bl/6 (Jackson Laboratory; 25-35 g, 8-20 weeks old at start of experiments) male and female mice were used for all experiments.
Wild animals	No wild animals were used.
Reporting on sex	Experiments have been performed in male and female mice. Sex of the animals is reported in the figures, figure legends and/or statistics table.
Field-collected samples	No field-collected samples were used.
Ethics oversight	All procedures complied with the animal care standards set forth by the National Institutes of Health and were approved by University of California, Berkeley's Administrative Panel on Laboratory Animal Care.

Note that full information on the approval of the study protocol must also be provided in the manuscript.
